# Bad medicine

**DOI:** 10.7554/eLife.00351

**Published:** 2012-12-13

**Authors:** Richard Smith

**Affiliations:** richardswsmith@yahoo.co.uk

**Keywords:** ben goldacre, big pharma, medicine, clinical trial

## Abstract

In his new book Ben Goldacre argues that the pharmaceutical industry is in poor health and in urgent need of treatment. **Richard Smith** agrees.

**BAD PHARMA**

**How drug companies mislead doctors and harm patients**

By Ben Goldacre

(Fourth Estate, 2012)

Reviewed by Richard Smith

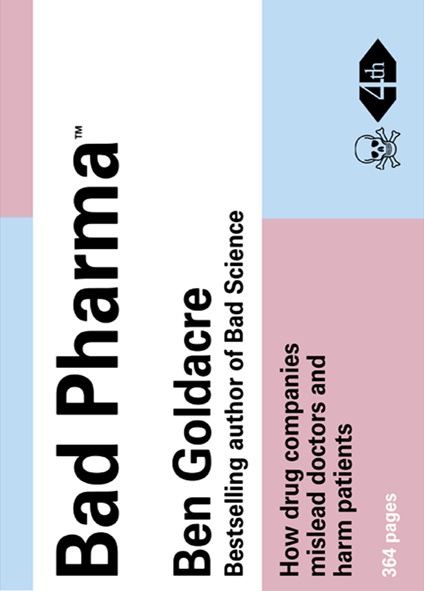

Ben Goldacre, a youngish medical doctor, made his name with a column called *Bad Science* in the *Guardian*, Britain's left leaning newspaper. Later he published a book with the same title that I've always thought of as a popular, funny, and highly readable textbook of clinical epidemiology. It was filled with villains corrupting science for their own dubious, usually financial, ends. Goldacre sliced them apart, and in doing so created a book that sold over 400,000 copies and was translated into 25 languages. Now he has published *Bad Pharma*, a darker, angrier book that amounts to a ‘j'accuse’ aimed primarily at the pharmaceutical industry but also at all of medicine. Although some people in the pharmaceutical and medical establishments have described the book to me as being ‘a bit over the top’, it is, in my opinion, a carefully constructed and convincing argument that the way we regulate, market and prescribe drugs is badly broken.

One story captured for me the discomfort and anger that Goldacre clearly feels. Earlier in his career he worked as a psychiatrist, and he describes prescribing reboxetine, an antidepressant, to a patient who had not responded to other drugs. Conscientiously he read the trial data and found only ‘well designed, fair tests with overwhelmingly positive results.’ The drug had been approved by the Medicines and Healthcare products Regulatory Agency (MHRA), the body that regulates new medicines in the UK, and had already been prescribed to millions of patients around the world. Goldacre and the patient discussed the evidence, and he wrote the prescription.

But both had been misled. A study published in October 2010 tracked down all the trials of reboxetine and found seven trials in which the drug had been tested against placebo (Eyding et al., 2010). Only one of the seven showed the drug to be better than placebo, and this study was the only one to be published. The six negative trials, which contained 10 times as many patients as the one positive trial, were not published. And of the trials that compared reboxetine with other drugs, only those that showed reboxetine to be as good as any other drug were published. A total of 507 patients were involved in these trials. However, the results of other trials (involving a total of 1657 patients) showed reboxetine to be worse than other drugs, and these were not published. Worse, the unpublished data showed that compared with patients taking other antidepressants, patients taking reboxetine were more likely to have side effects and to stop taking the drug.

Goldacre, a self-confessed nerd who believes passionately in the power of science and evidence, had been taken for a fool—along with millions of other doctors and patients. Almost a quarter of *Bad Pharma* is devoted to missing data, and it will be obvious to everybody that if we base our conclusions on only a biased sample of the evidence, then we will consistently draw false conclusions. Sadly the evidence is overwhelming and irrefutable that many trials are never published and that there is a systematic bias towards positive results. The consequence is that we believe drugs to be more effective and safe than they actually are.

Publication bias is a substantial problem throughout science. For example, a paper published in *Nature* earlier this caused much consternation by showing that of 53 early laboratory studies of promising targets for treating cancer, only six could be replicated (Begley and Ellis, 2012). The reason, suggested the authors of the study, is that fluke results that happen to be exciting are much more likely to be submitted to journals than boring, negative ones. We also know that the more exciting the result, the more likely it is to be published in a high-impact journal, with the unfortunate corollary that it is more likely to be wrong (Young et al., 2008). This means that while journals are supposed to help with information overload, because the highest impact journals publish the most important results, they are actually systematically misleading us.

Ironically we might hope that publication bias is much less of a problem with pharmaceutical studies than other academic studies because there are registers of trials, which is not the case for observational and others sorts of studies, and because pharmaceutical companies have to submit all their data to regulators to get their drugs licensed. Unfortunately, many trials continue to be published that have not been registered and, as Goldacre points out, most drugs in use today were launched long before registers of trials were introduced.

The fact that all data are supposed to be given to regulators does not solve the fundamental problem of missing data for a number of reasons, all of which are explained at length by Goldacre. First, information is often withheld from regulators. Second, data are given to the regulator in secret. Regulators will insist that they are fully competent to interpret the data, but I agree with Goldacre that in science we need to know how decisions are made and that many thousands of eyes scrutinising evidence are much better than a handful—which is why I've always insisted that post-publication peer review is the ‘real peer review’ (Smith, 2010). Third, regulators make it hard to access the data that they do have, and they also withhold study reports. This is unfortunate because drugs are not good or bad, they are somewhere on a spectrum between these extremes. Moreover, although a drug might not be right for some people, it will be appropriate for others—not least because patients have different values. The paternalistic process of regulators is surely out-dated.

Every chapter—indeed, every section—of *Bad Pharma* concludes with a list of what should be done (including by you, the reader), and the remedies for the problem of missing data include treating the withholding of data as professional misconduct, and ensuring that ‘the results of all trials included on humans must be made available within one year of completion, in summary table form if academic journal publication has not occurred.’ These remedies should apply to all research, and, as Goldacre acknowledges, we need to move beyond the publication of summary results of trials to the publication of the results on individual patients (anonymised, of course).

As Goldacre repeats several times in the book, missing data is the biggest barrier to the rational prescribing of drugs, but there are many others. The chapter on bad regulators (bad, as you can see, is Goldacre's favourite word) discusses the many conflicts of interest faced by regulators, and how they are reluctant to take drugs off the market once they have been approved. The biggest problem with regulation, which is the fault of legislators rather than regulators themselves, is that manufacturers are required simply to show that their drug is better than placebo, not that it is better than the standard treatment (which might not even be a drug). Increasingly, however, countries are overcoming this problem by having a second stage of technology assessment. In England, for example, the National Institute for Health and Clinical Excellence (NICE) assesses the value of new drugs, and only those that provide value for money are used by the National Health Service.

Missing data is the biggest barrier to the rational prescribing of drugs, but there are many others.

Unfortunately there are many ways to manipulate the result of trials, and we know that trials funded by industry are much more likely to come up with results that are favourable to the sponsor than trials that are publicly funded. One of the most memorable articles we published in the *BMJ* when I was editor was by two of the godfathers of evidence-based medicine, David Sackett and Andrew Oxman, who outlined how their spoof company HARLOT PLC worked: HARLOT stood for How to Achieve positive Results without actually Lying to Overcome the Truth, and its owners outlined 13 ‘honest’ ways to get the result you want from a trial (Sackett and Oxman, 2003). Goldacre describes many of the methods in his chapter on bad trials, beginning with ‘outright fraud,’ which may well be the least common. We know, based on conventional scores for the reporting of trials, that trials sponsored by drug companies are of higher quality than other trials, probably because they have more resources and are more careful in following guidelines set by regulators.

You can get the results you want by doing trials that are too small or too short; by testing your drug against either too low a dose or too high a dose of your competitor's drug; by stopping trials either to early or too late; by changing the outcome measures; by ignoring drop outs; by undertaking ‘dodgy subgroup analyses’; and by many other methods. Goldacre does a good job of explaining these highly technical processes, and those unfamiliar with the complexities of trials will surely be appalled that so many trials are unreliable.

Pharmaceutical companies now spend more money on marketing than on research, and Goldacre ends his book with a 100-page chapter on the marketing methods used by the pharma industry, many of them underhand. The industry spends $30–40 billion a year on marketing in the US, most of it aimed at doctors, and it pays for most of postgraduate education received by doctors. However, this money also buys, and this is not too strong a word, key opinion leaders, patient groups, journals, ghostwriters and doctors. Two past editors of the *New England Journal of Medicine*—Marcia Angell and Jerry Kassirer—have both published books on this theme (Kassirer, 2004; Angell, 2005).

Pharmaceutical companies now spend more money on marketing than on research.

Goldacre also has a message for doctors in the UK: if they continue to enjoy excessive largesse from drug companies because they regard this as normal, they risk following members of parliament (some of whom considered it normal to fiddle their expenses) and tabloid journalists (ditto for phone hacking) into disgrace. The US has introduced the Sunshine Act, which will require companies to name doctors to whom they have given money, and to say how much they gave and when. All countries need a similar act.

Like a good debater, Goldacre ends his book by answering the arguments that will be used against him. He has, critics will say, cherry picked examples; but he hasn't—he has used systematic reviews to make his case and anecdotes to make the book interesting. The pharma industry has guidelines, they will argue, and in any case, the bad behaviour that Goldacre describes is all in the past. The arguments are also easy to counter—the pharma industry sometimes flouts its own guidelines, and no, it isn't all in the past.

This is an important book. It perhaps won't sell as well as *Bad Science* because it isn't as entertaining, but it's a more important book in that it makes a powerful case of how ‘medicine is broken’. We need now a serious discussion of its evidence and its solutions. I hope that this book might lead to real change, but there is a sense of it being David against Goliath. But it was, of course, David who won.
